# An Event Recognition Method for Φ-OTDR Sensing System Based on Deep Learning

**DOI:** 10.3390/s19153421

**Published:** 2019-08-04

**Authors:** Yi Shi, Yuanye Wang, Lei Zhao, Zhun Fan

**Affiliations:** Guangdong Provincial Key Laboratory of Digital Signal and Image Processing, School of Engineering, Shantou University, Shantou 515063, China

**Keywords:** Φ-OTDR, event recognition, deep learning, convolutional neural network

## Abstract

Phase-sensitive optical time domain reflectometer (Φ-OTDR) based distributed optical fiber sensing system has been widely used in many fields such as long range pipeline pre-warning, perimeter security and structure health monitoring. However, the lack of event recognition ability is always being the bottleneck of Φ-OTDR in field application. An event recognition method based on deep learning is proposed in this paper. This method directly uses the temporal-spatial data matrix from Φ-OTDR as the input of a convolutional neural network (CNN). Only a simple bandpass filtering and a gray scale transformation are needed as the pre-processing, which achieves real-time. Besides, an optimized network structure with small size, high training speed and high classification accuracy is built. Experiment results based on 5644 events samples show that this network can achieve 96.67% classification accuracy in recognition of 5 kinds of events and the retraining time is only 7 min for a new sensing setup.

## 1. Introduction

Distributed optical fiber sensing technology refers to that multiple sensing units distributed on the same transmission optical fiber and the signals are collected through one channel. The phase-sensitive optical time domain reflectometer (Φ-OTDR) is a typical distributed sensing system which has a wide range of applications, including safety monitoring for important areas, pipeline monitoring and submarine power cable monitoring [1,2,3,4]. Researchers paid a lot of attention on the dynamic range, spatial resolution, and sensitivity of this system [5,6,7]. However, the ability of event recognition is always being the bottleneck limiting its performance in field application [8]. The complex environmental interference and harmless artificial disturbance may cause false alarms, resulting in a high nuisance alarm rate. Some methods based on signal feature extraction are proposed to recognize the event. Vries J et al. [9] extracted signal features from frequency domain, Jiang et al. [10] extracted the features through wavelet decomposition, Min et al. [11] extracted features through Gauss mixture model, Zhu et al. [12] used the level of cross rates of disturbance signal as the feature, Jiang et al. [13] chose the Mel-frequency sepctrum coefficients as the features and Zhang et al. [14] used multiple features for classification. These feature-based methods can achieve good recognition rate, but they need a careful selection of features and a relatively complex processing to extract these signal features. Besides, due to the coherent-fading problem, the disturbance shows a very weak appearance in certain sensing points, and the feature-based methods need to avoid using the signals from these fading points to extract features. In fact, these methods only use one temporal sensing signal for classification. However, different disturbances also cause different influences in spatial domain. Sun et al. [15] proposed an event recognition method based on morphologic features extracted from temporal-spatial data matrix and classified three kinds of events (artificial digging, walking and vehicle passing) through relevance vector machine. This work shows that the temporal-spatial data matrix supplies more information, and it is more suitable for event recognition. However, the processing of extracting morphologic features is complex and cannot be real-time.

In field application, the backscattered Rayleigh light is sensitive to various uncontrollable parameters, such as environment temperature, fiber installation condition and laser. It’s hard to find common signal features or models for classification. Traditional classification algorithm is dependent upon specific features or rules, but deep learning is different. It does not need to be provided the features or rules for classification [16]. It can spontaneously summarize rules from a large amount of input data and adjust its own parameters adaptively. Therefore, convolutional neural networks (CNN) based deep-learning is quite suitable for Φ-OTDR event classification. Some researches focused on event recognition in distributed optical fiber sensors using neural networks have been reported [17,18,19,20,21,22]. M. Aktas et al. [17] and L. Shiloh et al. [18] reported the high potential of deep learning in data processing for Φ-OTDR system in 2017 and 2018. Huijuan Wu et al. [19] reported a 1-D CNN for pipeline intrusion events detection, whose input is the denoised 1-D signal. S.Liehr et al. [20] reported a real-time optical fiber strain sensor using artificial neural networks, which shows the real-time advancement. Shallow CNN is unable to extract enough features from complex data adaptively, thus deep network is required. However, the deepening of the network structure will inevitably lead to the sharp attenuation of training speed, and easy to cause over-fitting. At the same time, the deep network has higher requirements on hardware, and the trained model will become larger. This is a serious obstacle for future efforts, such as porting to mobile devices. Therefore, the CNN used for event classification in Φ-OTDR system should have suitable depth and run fast enough for real-time.

This paper proposes a classification method based on CNN directly using temporal-spatial data matrix for Φ-OTDR system. This method does not need complex data processing before classification. Only a simple bandpass filtering and a gray scale image transformation are needed as pre-processing. A new structure of CNN with high classification accuracy, small size and high training speed is proposed, which is suitable for retraining for different field applications. Five types of events have been tested and the sensing data are collected by a home-made Φ-OTDR system. The result shows that this new CNN can achieve 96.67% classification accuracy in a 5644 sample data set and achieve a training speed of 35.61 steps/s with the Nvidia GPU (Titan X) which has 3854 computer unified device architecture (CUDA) cores, and the speed is almost 7 times faster than Inception-v3 network.

The Section 1 of the paper gives the introduction and research review. The Section 2 introduces how the data collection is established. The Section 3 shows the performance of traditional CNNs and how the optimized network is obtained. The Section 4 shows the performance of the optimized network and its comparison with Inception-v3. The Section 5 is the conclusion.

## 2. Data Collection

### 2.1. The Distributed Optical Fiber Sensing System

**Figure 1:**
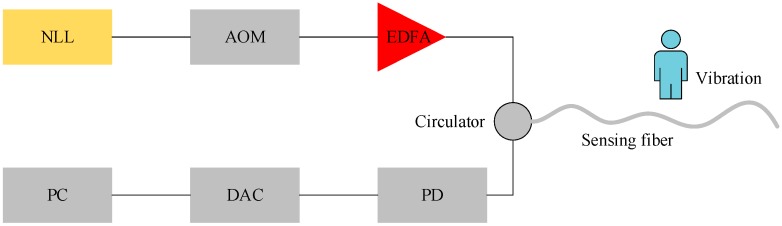
The distributed optical fiber sensing system.

The setup of home-made Φ-OTDR system is shown in Figure 1. An Ultra Narrow Linewidth Laser (NLL) with 3 kHz frequency width is used as the light source. An Acoustic Optic Modulator (AOM) shift chops the continuous light into probe pulses. An Erbium Doped Fiber Amplifier (EDFA) is used to compensate the light power loss. The amplified probe pulses are injected into the sensing fiber through a circulator. The Rayleigh backscattered (RBS) light wave is directly routed to a Photoelectric Detector (PD). The intensity evolution versus time is then recorded by a Data Acquisition Card (DAC) with 50 MHz sample frequency and processed in a computer (PC). The sensing fiber is G652 single mode fiber, with about 1 km, and buried five centimeters below the earth surface. Five types of event, which are background (No. I), walking (No. II), jumping (No. III), beating with a shovel (No. IV) and digging with a shovel (No. V), are applied at the same position of sensing fiber. In order to test whether the coherent fading condition will affect the event classification, two probe pulse width, 100 ns and 200 ns, are applied. The pulses repeat at a rate of 20 kHz. The data collected under two different pulse widths but with the same event type are treated as the same kind of data. The number of each type of event data is shown in Table 1.

Here are the five events in detail:

I. Background
  Instead of artificially adding disturbance, just collecting the noise of the environment.
II. Walking
  One person walks near the sensing fiber. The walking speed is about 1.2 m per second.
III. Jumping
  One person jumps near the sensing fiber at a rate of about once a second.
IV. Beating with a shovel
  One person takes a shovel to tap earth surface near the sensing fiber at a rate of about once a second.
V. Digging with a shovel
  One person takes a shovel to dig near the sensing fiber at a rate of about once a second.

### 2.2. Data Pre-Processing

**Figure 2:**
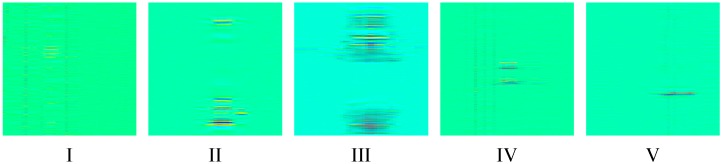
The data matrix after bandpass filter.

Taking each of the Rayleigh backscattering traces as a row forms the data matrix. The horizontal row of the data matrix stands for space domain and the vertical column of the data matrix stands for time domain. The light intensity from each scattering positions are different, leading to different direct current (DC) component intensity in time domain. Thus, a bandpass filtering is applied to remove the DC component in each column. The pass band is set to be 5 Hz to 15 kHz. The typical temporal-spatial data matrixes of each event after bandpass filter are shown in Figure 2.

**Figure 3:**
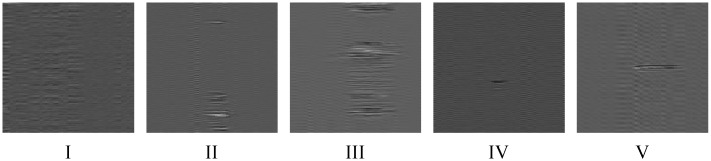
The typical gray image of each event type.

Each matrix stands for 50 m spatial length and 1 s temporal length. Before sending these matrixes to CNN, each matrix is turned to be a gray scale image and adjusted the size to 229 × 229. The typical gray images of each event are shown in Figure 3.

## 3. Event Recognition

### 3.1. Comparison of Common CNNs

Usually, the capability of classification of CNN is proportional to the depth. However, the deep network may cause a serious decrease of training speed and cause over fitting problem. Thus, a suitable network structure is important. Some common CNNs, such as LeNet [23], AlexNet [24], ResNet [25], VggNet [26], GoogLeNet [27], are tested firstly. LeNet is the first mature CNN, specially designed to deal with the classification of MNIST digital character set. AlexNet is deeper than LeNet, specially designed to deal with the classification of 224 × 224-sized colour pictures. VggNet is deeper than AlexNet, but no longer uses convolution kernel of large size. GoogLeNet is a network structure based on network in network. ResNet puts forward the idea of residual learning. The training set and the validation set in Table 1 are used for training and testing the performance of each CNN. The training parameters of all the CNNs are the same. The learning rate is 0.01, total training steps are 50,000 and the optimizer is root mean square prop (RMSProp) [28]. The results are shown in Table 2.

From Table 2, VggNet and GoogLeNet achieve better classification accuracy (>95%) than other models. Considering that VggNet is much bigger and its training speed is slower than GoogLeNet, GoogLeNet is chosen to be the basic CNN structure.

### 3.2. Optimization of CNN

GoogLeNet can achieve good classification accuracy. However, it is a huge network with a relatively low training speed. As the CNN need to be retrained for every new setup of sensing fiber, a smaller and faster network which can still keep the accuracy is needed.

**Figure 4:**
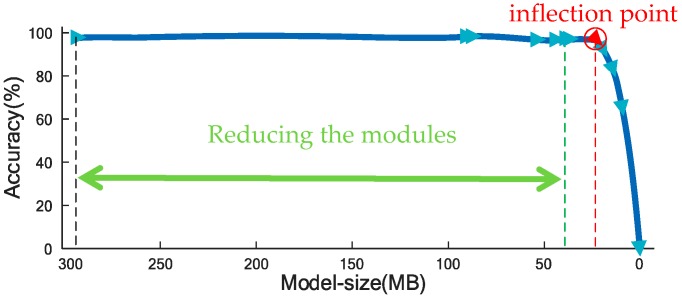
The relationship between model size and classification accuracy.

**Figure 5:**
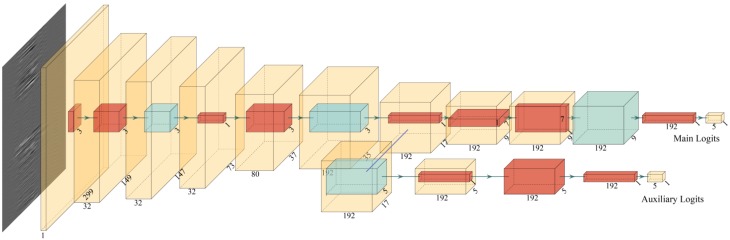
The optimized network structure (the red cube denotes convolution operation and the blue cube denotes pooling operation).

Inception-v3 of GoogLeNet is chosen to be the original basic network. There are many inception modules in Inception-v3 structure [27]. These repeated modules with similar structure are firstly removed one by one until only one module remained (the green line in Figure 4). Then the parallel paths in the last remained inception module are reduced one by one until only one path remained (the red line in Figure 4). Then reduce each convolutional layers one by one. After each reducing step, the total model size is measured, a classification procedure (the same as in Section 3.1) is applied and the classification accuracy is obtained. The relationship between the model size and the classification accuracy is figured out and shown in Figure 4. From Figure 4, it can be observed that the classification accuracy basically keeps the same when the model size is larger than 24 MB. This means most of the inception modules have similar function and only one module is essential. There is an inflection point in Figure 4, which denotes the least size of CNN structure. Based on analyzing the relationship between accuracy and model size, a new CNN structure, which is as small as possible, is proposed and shown in Figure 5 in detail. In Figure 5, the red cube denotes convolution operation and the blue cube denotes pooling operation.

In Figure 5, the 299 × 299-sized gray image of event is used as the input. The gray image firstly performs 2-dimentional convolution with step of 2 points with 32 3 × 3 × 1 kernels and a feature matrix with dimensions 149 × 149 × 32 is obtained. Following anther convolution with 32 filters with dimensions 3 × 3 × 32, a feature matrix with dimensions 147 × 147 × 32 is obtained. The next pooling operation performs a maximum operation in a 3 × 3 sized kernel with step of 2 points and a feature matrix with dimensions 73 × 73 × 32 is obtained. Following is another two convolutional layers and one pooling layer. After these convolution and pooling, the features of the gray image are extracted. Then there are two parallel paths after the 7th layer. One is the main output path and another is an auxiliary output path. In the main path, three convolutional operations are performed first and one average pooling operation follows. The main outputs are obtained by a final convolution operation with five 1 × 1 × 192 kernels, instead of traditional fully connected layer. This replacement will help reduce the size of the network. In the auxiliary path, one average pooling operation are performed first and then three times convolutional operation. The auxiliary outputs are also obtained by a convolution operation with five 1 × 1 × 192 kernels. The five output logits in main outputs and auxiliary outputs denotes the five kinds of events.

The auxiliary path here is to help the training process of CNN. In this task, the features produced by the 7th layer are very discriminative. By adding the auxiliary path connected to this layer, it may help encourage the final classifier and increase the back-forward gradient [29]. During training, the loss from auxiliary logits is added to the loss of main logits by a weight of 0.3. At the inference time, only the main path is applied.

## 4. Analysis of Classification Performance

**Figure 6:**
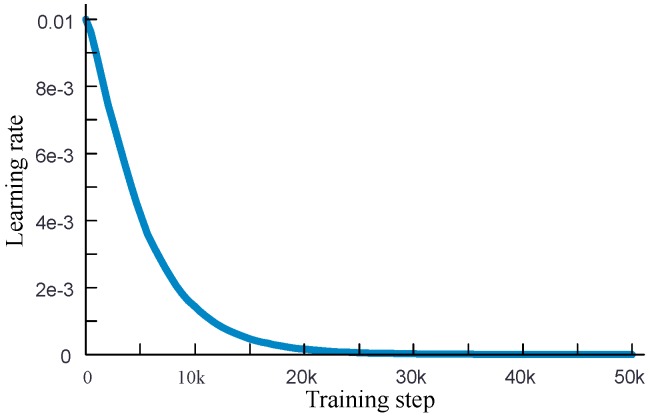
The learning curve of training.

**Figure 7:**
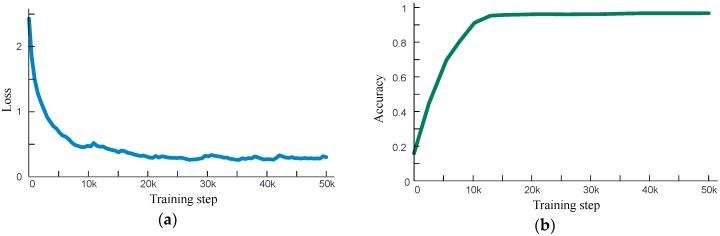
Loss curve (**a**) and classification accuracy curve (**b**) of training.

The network shown in Figure 5 is applied for classification. An exponential attenuation learning rate with initial value of 0.01, which is shown in Figure 6, is applied for accelerating the training. The training data is the 4515 gray images of 5 kinds of events, shown in Table 1. The total training step is set to be 50,000 and the optimizer is RMSProp. The batch size of training data is 32, and training with the GPU with 3854 CUDA cores. The loss curve and accuracy curve are shown in Figure 7. Figure 7 shows that the network can converge and achieve 95% accuracy after 16,000 training steps.

**Figure 8:**
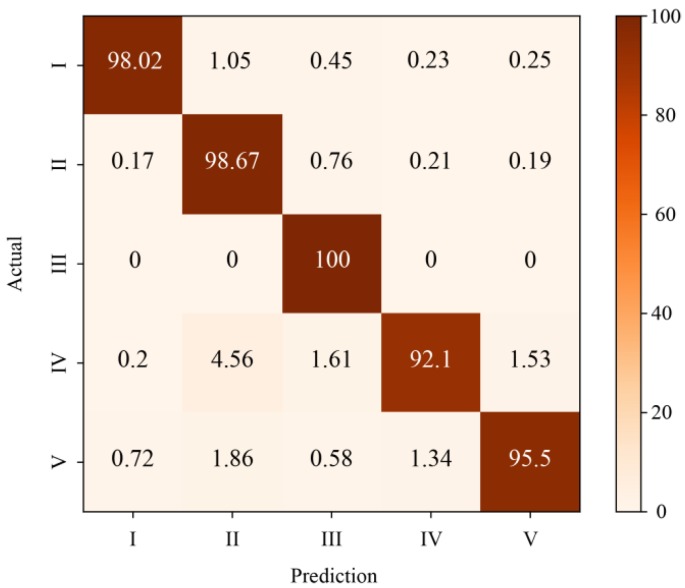
Confusion matrix of five events’ classification.

1129 gray images of 5 kinds of events are used for validation. The result of classification is shown in Table 3 and the confusion matrix is shown in Figure 8. For background (No. I), walking (No. II) and jumping (No. III), the CNN model can achieve more than 98% accuracy. About 7.9% of beating with shovel events (No. IV) and 4.5% of digging with shovel (No. V) are missed. Jumping (No. III), beating with shovel events (No. IV) and digging with shovel (No. V) all show an impact type of signal, which is easy to cause confusion. In top 2 accuracy, almost 100% classification accuracy can be achieved except for 1% missing in beating with shovel event (No. IV).

**Figure 9:**
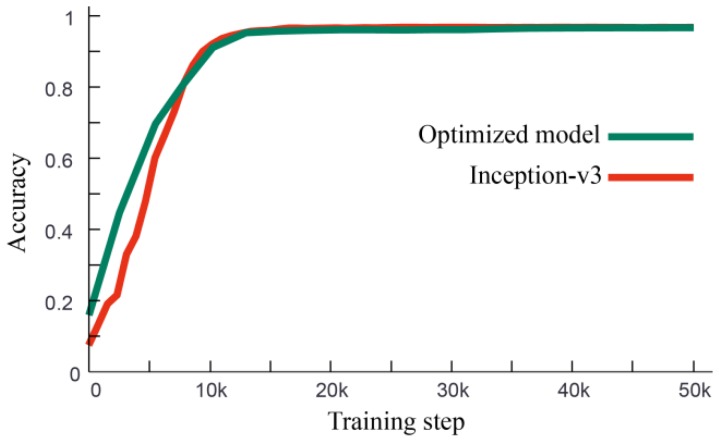
Accuracy curve of optimized network (green) and Inception-v3 (red).

A comparison between the optimized model in Figure 5 and original Inception-v3 has also been carried out. With the same training data, training parameters and validation data, the training processing and the performance of these two networks are shown in Figure 9 and Table 4, respectively. It can be seen that the final accuracy of the optimized model is 96.67% and for Inception-v3, it is 97.08%, which is close. Both of the two networks can achieve 95% classification accuracy after 16,000 steps training. But the training speed of the optimized model is 35.61 steps/s, which is almost 7 times faster than the training speed of Inception-v3. As the essential training steps are 16,000, it only needs about 7 min to retrain this CNN for a different field application.

## 5. Conclusions

This paper has proposed an event recognition method for Φ-OTDR distributed optical fiber sensing system based on deep learning. The temporal-spatial data matrix from Φ-OTDR is directly applied as the input of CNN. Only simple bandpass filtering and gray image transformation are carried out as the pre-processing. Based on analyzing the common-used CNNs, a small network structure is proposed with high classification accuracy and fast training speed. This fast network is very suitable for retraining for different field applications. Field experiment of five kinds of events has been carried out. The results show that the coherent fading phenomenon in Φ-OTDR will not affect the classification and the proposed network can achieve 96.67% classification accuracy with a 35.61 steps/s training speed.

## Figures and Tables

**Table 1:** The number of every type of data.

Event Type	I	II	III	IV	V
Training Set	307	1122	1101	1237	748
Validation Set	77	280	275	310	187
Total Number	384	1402	1376	1547	935

**Table 2:** The performance results of common CNNs.

Model Name	Model Size (MB)	Training Speed (step/s)	Classification Accuracy (%)	Top 2 (%)
LeNet	39.3	90.9	60	86.5
AlexNet	554.7	19.6	94.25	99.08
VggNet	1638.4	2.53	95.25	100
GoogLeNet	292.2	4.1	97.08	99.25
ResNet	282.4	7.35	91.9	97.75

**Table 3:** The classification accuracy of five events.

Type of Accuracy	I	II	III	IV	V
Accuracy (%)	98.02	98.67	100	92.1	95.5
Top 2 accuracy (%)	100	100	100	99	100

**Table 4:** Performance comparison with Inception-v3.

Network	Accuracy (%)	Top 2 Accuracy (%)	Training Speed (steps/s)	Model Size (MB)
The optimized network	96.67	99.75	35.61	20
Inception-v3	97.08	99.25	4.35	292.2
